# Understanding the clinical genetics of kidney stone disease using the Natera Renasight panel

**DOI:** 10.1007/s00240-025-01723-2

**Published:** 2025-03-24

**Authors:** Andrewe L. Baca, Rutul D. Patel, Kevin Labagnara, Benjamin Green, Michael Zhu, Kavita Gupta, Beth Edelblute, Andrea A. Asencio, Deep Sharma, Wei Chen, Dima Raskolnikov, Jillian Donnelly, Kara L. Watts, Alexander C. Small

**Affiliations:** Departments of Urology and Nephrology, Montefiore Medical Center, Albert Einstein College of Medicine, 1250 Waters Pl, Tower 1 Penthouse, Bronx, NY 10461 USA

**Keywords:** Kidney stones, Kidney diseases, Genetic testing, Urology, Nephrology

## Abstract

We aimed to characterize the underlying genetics of kidney stone disease (KSD) in an urban and diverse population using the Natera Renasight genetic panel. This was a single-center prospective study of high-risk KSD patients, defined as recurrent stone formers or those with a family history with KSD. Buccal saliva DNA samples were collected with the commercially available Natera Renasight genetic panel and were analyzed using next-generation sequencing. The panel assesses 385 kidney disease related genes, including 45 linked to KSD. One hundred eleven high-risk KSD patients were enrolled. The majority were female (56%) with a median age of 50 (IQR 39.5–59.5), compromising a diverse ethnic background with 62% Hispanic, 23% White and 11% Black. Patients had median 3 (IQR 2–5) lifetime stone episodes, and 41% had family history of KSD. Genetic analysis was possible for 105 patients (95%). Eight (8%) had positive tests with only one patient found to have a pathogenic mutation associated with KSD (SLC7A9, cystinuria). The other 7 positive tests included amyloidosis (TTR, N = 3), Alport syndrome (COL4A3, N = 2), polycystic kidney disease (PKD1, N = 1), and susceptibility to ESRD (APOL1, N = 1). Patients with positive tests were more likely to have chronic kidney disease (38% vs 5%, p < 0.01), gout (13% vs 1%, p = 0.02) and carbonate apatite stones (38% vs 7%, p < 0.01). Our study sheds light on genetic factors of KSD in a diverse patient population. The results suggest that KSD is unlikely monogenetic in nature, but is more likely due to a complex interplay of polygenetic and environmental influences. Genetic testing may be most useful in KSD patients with chronic kidney disease.

**Supplementary Information:**

The online version contains supplementary material available at 10.1007/s00240-025-01723-2.

## Introduction

Kidney stone disease (KSD) exhibits a multifactorial etiology with increasing recognition of its genetic link [1]. Twin studies have shown that heritability accounts for 45% to 56% of kidney stone prevalence [2, 3]. A landmark study involving 272 kidney stone patients demonstrated that 15% of patients had monogenetic disease-causing mutations among 14 genes, with a higher prevalence in pediatric (21%) compared to adult (11%) patients [4]. Genome-wide association studies have identified additional mutations linked to KSD [5, 6].

The integration of genetic testing into clinical practice is on the rise. While the 2019 American Urological Guidelines for KSD do not specifically recommend genetic testing [7], The International Alliance of Urolithiasis (IAU) guidelines state that “Genetic testing can aid in the diagnosis of monogenic urinary stone diseases.”[8] The Natera Renasight genetic test has the ability to detect kidney disease related mutations on 385 genes including 45 associated with KSD.[9] We aimed to characterize the underlying genetics of KSD in an urban and diverse population using the Natera Renasight panel.

## Methods

We conducted a single-center observational study to evaluate the genetics of patients with KSD presenting to outpatient urology and nephrology clinics at an academic medical center. The study was approved by the Institutional Review Board (IRB #2021–12872). We included adult patients (≥ 18 years) with a personal history of kidney stones (≥ 2 episodes in the past 5 years), a personal history of stone(s) with a family history of KSD, or those with a metabolic workup indicative of a predisposition to KSD, such as abnormal 24-h urine studies or serum chemistry results. Exclusion criteria included prior genetic testing for KSD, inability or unwillingness to comply with study assessments, acute stone passage, active urinary tract infection, pregnancy, renal transplant recipients, urinary tract anatomical anomalies, and use of stone-forming medications (e.g. Indinavir, Sulfamethoxazole, Amoxicillin).

Participants underwent genetic testing using the Natera Renasight test (Natera Inc, TX), which screens for 385 kidney disease-related genes, including 45 specifically associated with KSD (Appendices 1 and 2). The Renasight test kits were provided by Natera for the purposes of this study at no cost to the patient. The test was conducted on buccal saliva swab samples that were anonymized before being shipped to the lab for analysis. Full gene sequencing was performed via next-generation sequencing (NGS) and copy number variant (CNV) analysis. Confirmation was done via Sanger sequencing or MPLA/qPCR for CNVs. NGS testing was done via hybrid capture enrichment methods using clinical exome backbone baits. The average depth across the region of interest for the panel is ~ 170x. The minimum coverage for curated variants is 8x.

Genetic reports are categorized into “positive”, “carrier”, or “negative”. Patients may also have “variants of unknown significance (VUS).” A positive result indicates that presence of one or more mutations associated with disease. Carrier results identify individuals with one mutation linked to autosomal recessive conditions but are not affected. Negative results show no known disease-causing variants. VUS denotes DNA mutations of uncertain significance, often representing normal human variation. Follow-up telehealth visits occurred 4–8 weeks after enrollment to review genetic results. Genetic counseling was emphasized and offered for those with positive or carrier test results.

Patient demographics and clinical histories were abstracted from medical records, focusing on comorbidities such as diabetes, CKD (EGFR < 60 mL/min/1.73 m^2^ calculated using the 2021 CKD-EPI equation), and recurrent urinary tract infections (rUTIs). Standard of care stone workup included blood and urine analyses, including serum electrolytes, intact parathyroid hormone (PTH), vitamin D levels, urine microscopic analysis, and stone composition via x-ray crystallography or infrared spectroscopy. Additionally, 24-h urine testing was performed using the LabCorp Litholink home test kit.

A power analysis estimated that sample size of 111 participants was necessary to detect an incidence of monogenetic stone-causing mutations with 80% power, using a baseline incidence of 11%[4] and assuming that would increase to 20% using a broader genetic panel. We assumed a 20% dropout rate for withdrawal of insufficient sample for analysis. Statistical analyses were conducted across key groups: comparing positive and negative genetic tests, evaluating KSD-associated genes (positive, carrier, or VUS) to those without, and assessing 24-h urine test results in patients with or without KSD-associated genes. Additionally genetic results were analyzed by disorders of calcium, uric acid, oxalate metabolism, and cystinuria. Chi-Square analysis and homoscedastic, two tailed T-tests were used to compare categorical and continuous variables respectively. Significance was set at p < 0.05.

## Results

There were 111 KSD patients consented for inclusion in this study. Characteristics of the study population are in Table 1. The majority were female (56%), and median age was 50 (IQR 39.5–59.5). Participants represented a diverse ethnic background with 62% Hispanic, 23% White and 11% Black. Patients had a median of 3 (IQR 2–5) lifetime stone episodes and 81% had prior stone surgery. 41% had a family history of kidney stones. Most patients had calcium oxalate (62%) or calcium phosphate (10%) stones. The most common comorbidities were diabetes (23%), recurrent urinary tract infections (11%) and CKD (7%).

**Table 1:** Participant Demographics and Renasight Results

	Total	Positive panel	Negative panel	P-value	Stone VUS	Other VUS	P-value
N	111	8	97		51	54	
Gender							
Female (%)	62 (56%)	5 (62%)	52 (54%)	0.63	30 (59%)	27 (50%)	0.36
Age, Median (IQR)	50 (39.5–59.5)	50 (39–55)	48 (39–59)	0.85	48 (36–57)	51 (42–61)	0.31
Race/Ethnicity							
Asian (%)	7 (6%)	1 (12%)	5 (5%)	0.39	4 (8%)	2 (4%)	0.36
Black (%)	12 (11%)	2 (25%)	9 (9%)	0.16	4 (8%)	7 (13%)	0.39
White (%)	25 (23%)	2 (25%)	22 (23%)	0.88	13 (25%)	11 (20%)	0.53
Other (%)	67 (60%)	3 (38%)	61 (63%)	0.16	30 (59%)	34 (65%)	0.66
Hispanic Ethnicity (%)	69 (62%)	4 (50%)	62 (64%)	0.32	32 (63%)	34 (63%)	0.98
Comorbidities							
CKD (%)	8 (7%)	3 (38%)	5 (5%)	0.0009	2 (4%)	6 (11%)	0.17
Hyperparathyroidism (%)	6 (5%)	0 (0%)	6 (6%)	0.47	3 (6%)	3 (6%)	0.94
Recurrent UTI (%)	12 (11%)	0 (0%)	12 (12%)	0.29	5 (10%)	7 (13%)	0.61
Gout (%)	2 (2%)	1 (13%)	1 (1%)	0.02	1 (2%)	1 (2%)	0.97
Stone history							
Family history KSD (%)	46 (41%)	5 (62%)	41 (42%)	0.27	20 (39%)	26 (48%)	0.36
Most recent stone size mm, median (IQR)	8 (5–12)	9.5 (4.75–11.25)	8 (5–12)	0.59	7 (5–11)	9.5 (6.25–12.75)	0.35
Lifetime stone episodes, median (IQR)	3 (2–5)	3 (2.75–5)	3 (2–5)	0.94	3 (2–5)	4 (2–5)	0.16
Spontaneous passage episodes, median (IQR)	1 (0–2)	1 (0–2.25)	1 (0–2)	0.80	1 (0–2)	1 (0–3)	0.15
Prior stone operations, median (IQR)	1 (1–2)	1.5 (0–2)	1 (1–3)	0.78	1 (1–2)	1.5 (1–3)	0.81
Required surgery (%)	90 (81%)	5 (62%)	80 (82%)	0.17	41 (80%)	44 (81%)	0.89
Stone prevention meds							
Potassium Citrate (%)	12 (11%)	1 (13%)	11 (11%)	0.92	4 (8%)	8 (15%)	0.26
Thiazide Diuretic (%)	10 (9%)	1 (13%)	9 (9%)	0.77	6 (12%)	4 (7%)	0.45
Both (%)	6 (5%)	0 (0%)	5 (5%)	0.51	1 (2%)	4 (7%)	0.19
Stone type							
Brushite (%)	3 (3%)	0 (0%)	3 (3%)	0.61	0 (0%)	3 (6%)	0.08
CaOx Dihydrate (%)	8 (7%)	0 (0%)	7 (7%)	0.43	1 (2%)	6 (11%)	0.06
CaOx Monohydrate (%)	61 (55%)	2 (25%)	55 (57%)	0.08	29 (57%)	28 (52%)	0.61
Carbonate Apatite (%)	11 (10%)	3 (38%)	7 (7%)	0.005	5 (10%)	5 (9%)	0.92
Struvite (%)	1 (1%)	0 (0%)	1 (1%)	0.77	1 (2%)	0 (0%)	0.30
Uric Acid (%)	9 (8%)	0 (0%)	9 (9%)	0.37	5 (10%)	4 (7%)	0.66
No Stone Analysis	18 (16%)	3 (37%)	15 (16%)	0.11	10 (20%)	8 (15%)	0.51

*VUS* Variance of Unknown Significance, *IQR*  Interquartile Range, *CKD* Chronic Kidney Disease, *UTI*  Urinary Tract Infection, *KSD* Kidney Stone Disease, *CaOx*  Calcium Oxalate

**Fig. 1:**
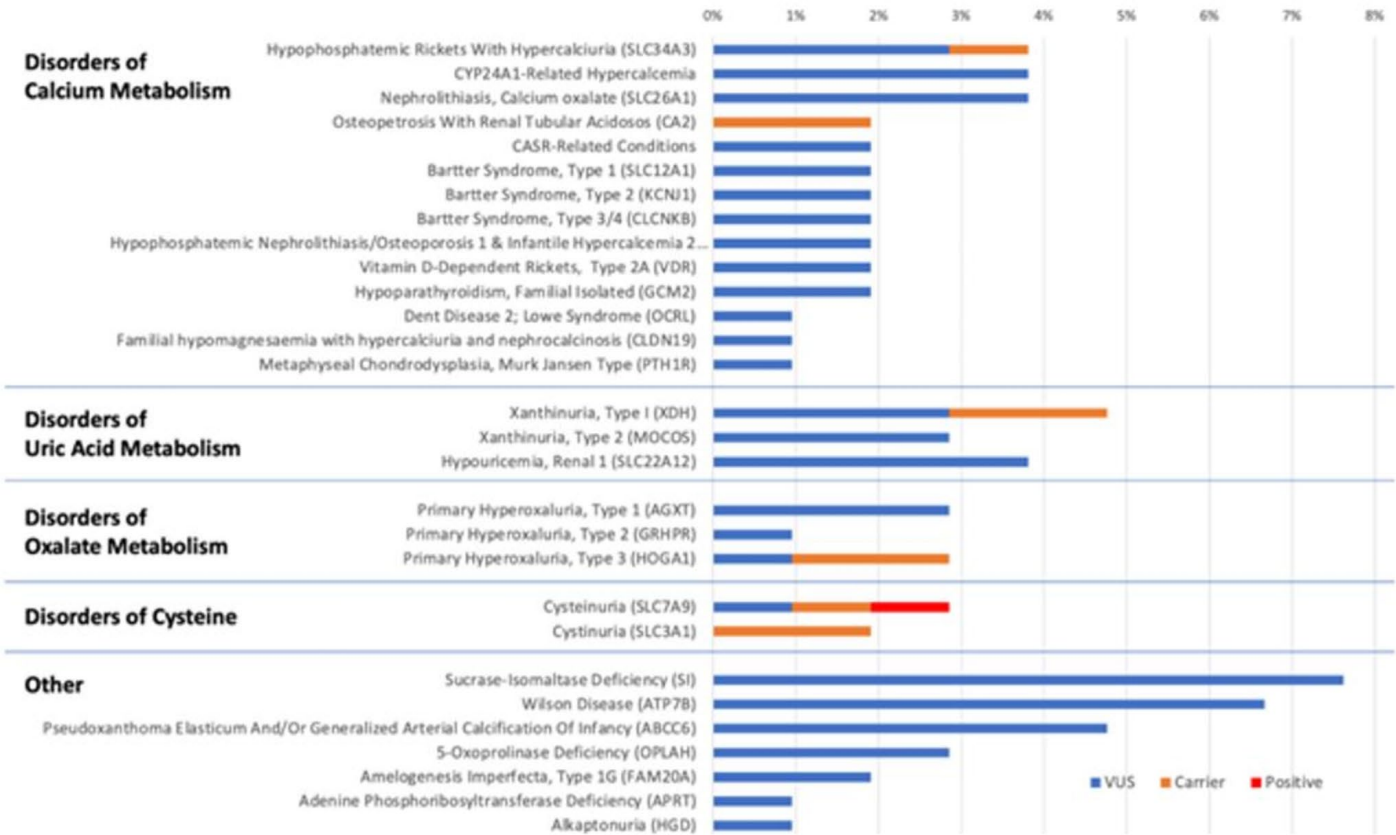
Genetic Frequency among 105 kidney stone disease patients on Natera Renasight Panel (Kidney Stone related genes only)

One hundred fivepatients (95%) provided samples sufficient for genetic analysis. The results of all KSD-related genes are summarized in Fig. 1. Eight (8%) patients had positive genetic test results. Only 1 had a KSD-associated autosomal dominant pathogenic mutation, which was for cystinuria (*SLC7A9*). Of note, this patient had no prior history of cystinuria and was known to form calcium phosphate stones. The 7 other positive tests included amyloidosis (*TTR*, N = 3), Alport syndrome (COL4A3, N = 2), polycystic kidney disease (*PKD1*, N = 1), and susceptibility to end stage renal disease (*APOL1*, N = 1). Patients with positive tests were significantly more likely than patients with negative tests to have chronic kidney disease (38% vs 5%, p < 0.01), gout (13% vs 1%, p = 0.02), or calcium phosphate stones (38% vs 7%, p < 0.01).

Forty-nine patients (47%) were carriers for 66 unique genes, of which 8 (8%) were carriers of KSD genes. The most common carrier genes detected were not KSD-associated, including susceptibility to end stage renal disease (*APOL1*, N = 16), congenital nephrotic syndrome type 2 (*NPHS2*, N = 6), sickle cell disease (*HBB*, N = 3), and methylmalonic aciduria (*MUT*, N = 3). Several carriers of KSD-associated genes were identified including cystinuria (*SLC3A1*, N = 2 or *SLC7A9*, N = 1), xanthinuria type 1 (*XDH*, N = 2), osteopetrosis with renal tubular acidosis (*CA2*, N = 2), primary hyperoxaluria type 3 (*HOGA1*, N = 2), and hypophosphatemic rickets with hypercalciuria (*SLC34A3*, N = 1).

All 105 patients had multiple VUS spanning 279 unique genes. Of those, KSD-associated VUS were noted in 56 patients (53%) on 30 unique genes. The most common VUS overall were not KSD-linked, including polycystic kidney disease (*PKD1*, N = 21), familial Mediterranean fever (*MEFV*, N = 17), Fraser syndrome (*FRAS1*, N = 11), familial hyperaldosteronism type IV (*CACNA1H*), and Senior-Loken syndrome 4 (*NPHP4*, N = 10). The most common KSD-associated VUS were sucrase-isomaltase deficiency (*SI*, N = 8), Wilson disease (*ATP7B*, N = 7), pseudoxanthoma elasticum and generalized arterial calcification of infancy (*ABCC6*, N = 5), *CYP24A1*-related hypercalcemia (*CYP24A1*, N = 4), and hypouricemia Renal 1 (*SLC22A12*, N = 4). There were no significant baseline differences between KSD-associated VUS patients and all other VUS patients.

Together, 56 patients (53%) had KSD-associated genetic findings across 33 genes, summarized in Fig. 1. Disorders of calcium metabolism were the most common, with 31 genetic results affecting 29 patients (28%). Disorders of uric acid metabolism had 12 genetic results on xanthinuria (*XDH* and *MOCOS*) and renal hyperuricemia (*SLC22A12*) genes, affecting 11 patients (10%). Disorders of oxalate metabolism were noted on 7 genetic results of primary hyperoxaluria genes (Type 1 *AGXT*, Type 2 *GRHPR*, and Type 3 *HOGA1*) in 7 patients (6%). Cystinuria genes were identified 5 times (*SLC7A9* and *SLC3A1*) in 5 patients (5%). Other KSD-associated genes were noted in 27 instances in 21 patients (19%).

Fifty patients (45%) completed 24-h urine testing for metabolic stone evaluation and 93 patients (88%) had stones available for analysis. Patients who completed 24-h urine testing had significantly different baseline characteristics from those who did not complete testing – they had more lifetime stone episodes (median 4, IQR 3–6 vs 2, IQR 2–4, p < 0.01), had more total stone procedures (median 2, IQR 1–3 vs 1, IQR 1–2, p = 0.03), and were more likely to take stone prevention medications (N = 24 vs N = 4, p < 0.01). Patients had similar urine profiles whether they had positive (N = 5) or negative (N = 45) genetic tests. Patients had similar urine profiles whether they had any KSD-linked genes (N = 25) or did not (N = 25), except for slightly higher levels of urine citrate (604 ± 300 vs 422 ± 320, p = 0.04). Among patients with genes related to disorders of calcium metabolism (N = 16), 24-h urine calcium levels were not significantly different from those without these genes (N = 38) (155 ± 90 vs 166 ± 83 mg/day, p = 0.66). They were also not more likely to have calcium oxalate stones (59% vs 63%, p = 0.65). Among patients with genes related to disorders of uric acid metabolism (N = 4), 24-h urine uric acid levels were not significantly different from those without these genes (N = 50) (0.46 ± 0.11 vs 0.68 ± 0.36 g/day, p = 0.23). They were more likely to have uric acid stones (27% vs 6%, p = 0.01). Among patients with genes related to disorders of oxalate metabolism (N = 2), 24-h urine oxalate levels were not significantly different from those without these genes (N = 52) (27 ± 8 vs 34 ± 13 mg/day, p = 0.52).

## Discussion

In this single-center prospective study, we found potential utility for genetic testing among a diverse cohort of kidney stone patients. Results indicated that 8% of patients exhibited positive genetic tests, notably featuring a single patient with a pathogenic mutation of SLC7A9 associated with cystinuria. Many patients were carriers or had VUS in genes associated with KSD. Interestingly, those with positive genetic tests were more likely to have CKD, gout, or form carbonate apatite stones. No significant differences were observed in 24-h urine parameters between patients with or without KSD-associated genes.

Currently, there are no clear guidelines regarding the role of genetic testing for KSD. Genetic testing is typically considered for patients with recurrent KSD, strong family history of KSD, pediatric-onset of KSD, unusual stone composition (e.g., cystine stones), or have persistently failed standard prevenative measures and treatment. This study highlights that genetic testing using the Natera Renastight genetic test kit might be useful for recurrent stone formers with CKD, gout, or carbonate apatite stones. Since the Renasight genetic test was originally designed for patients with CKD, it is probably most useful for patients with CKD. Although there was one positive test for a KSD related gene, SLC7A9 (Cystinuria), it did not change their management as they formed carbon phosphate stones and not cystine stones likely due to incomplete penetrance. Subsequently, there were no changes in the KSD management for the other seven patients with positive pathogenic tests on non-KSD related genes.

This study highlights the intricate genetic landscape of KSD, emphasizing the significance of considering genetic factors in patient management. Identifying specific pathogenic mutations is valuable for diagnosis and tailored treatment, while the prevalence of carriers and VUS underscores the multifaceted nature of the disease. Detection of specific genetic disorders, such as cystinuria, enables targeted interventions, potentially reducing recurrence and complications. Furthermore, genetic insights can refine screening protocols for associated conditions to facilitate early detection and prevention. Genetic counseling plays a pivotal role in elucidating test results and supports informed decision-making amongst patients and their families.

Halbritter et al. reported a high prevalence of monogenetic mutations in KSD patients, with pathogenic mutations found in 15% of cases (21% in pediatric and 11% in adult patients). In contrast, our study identified potential pathogenic mutations in only 1 out of 105 patients (< 1%). This discrepancy may reflect the broader inclusion criteria and more diverse population in our study. While monogenetic mutations play a role in KSD, their rarity in our study highlights the complexity of genetic factors contributing to stone formation. Our patient population had a notable prevalence of carriers and VUS in KSD-associated genes related to cystinuria, calcium, uric acid, and oxalate metabolism.

### Disorders of calcium metabolism

Genetic predispositions linked to KSD and nephrocalcinosis exhibit significant variability, often presenting with nuanced manifestations. In our investigation, 28% of patients manifested genetic variants associated with calcium metabolism, encompassing mutations in *CYP24A1*, hereditary hypophosphatemic rickets, and Bartter syndrome. *CYP24A1* mutations disrupt vitamin D metabolism, precipitating hypercalcemia [10]. Notably, VUS in *CYP24A1* were detected in 4% of patients. Hypophosphatemic rickets with hypercalciuria arises from *SLC34A3* gene anomalies, inducing phosphate excretion and hypercalciuria[11]; VUS in *SLC34A3* were identified in 4% of patients. Bartter syndrome, encompassing types 1–3, stems from mutations in genes such as *NKCC2* (*SLC12A1*), *ROMK* (*KCNJ1*), or CIC-Kb (*CLCNKB*), culminating in hypokalemic alkalosis and hypercalciuria[1, 12]. Notably, Bartter syndrome VUS were present in 6% of patients.

### Disorders of oxalate metabolism

Mutations in oxalate metabolism can contribute to calcium oxalate stones [13, 14]. Primary Hyperoxaluria encompasses three types, all autosomal recessive: Type 1 results from alanine-glyoxylate aminotransferase (*AGT*) deficiency, Type 2 involves glyoxylate reductase hydroxypyruvate reductase (*GRHPR*) deficiency, and Type 3 stems from 4-hydroxy-2-oxoglutarate aldolase (*HOGA*) deficiency [15]. Our study found primary hyperoxaluria genes in 6%. The role of variable penetrance of these disorders leading to oxalate overproduction is still being studied.

### Disorders of uric acid metabolism

Genetic disorders affecting uric acid metabolism can result in non-calcium kidney stone formation. Hereditary xanthinuria, classified as Type 1 or Type 2, stems from mutations impacting xanthine metabolism. Type 1 results from autosomal recessive mutations in xanthine dehydrogenase (*XDH*), and Type 2 involves mutations in molybdenum cofactor sulfurase (*MOCOS*) [16]. We found 2% were carriers and 3% had VUS associated with the *XDH* gene, and 2% had VUS associated with the *MOCOS* gene. Interestingly, one patient had 2 separate VUS within the same *MOCOS* gene (p.Gly573Glu and p.Met617Val). Incidence is reported as 1 in 69,000 but estimated at 1 in 6,000 due to asymptomatic cases [17]. Both types of xanthinuria lead to xanthine accumulation, triggering xanthine stones [1]. Hereditary hypouricemia, from *SLC22A12* mutations can cause uric acid stone formation due to impaired uric acid resorption [18]. We found 4% of patients had VUS associated with this gene. Management includes fluid intake, urinary alkalinization, and potentially xanthine oxidase inhibitors [19, 20].

### Cystinuria

Cystinuria is the leading cause of nephrolithiasis in children and is due to disrupted amino acid transporters in the proximal tubule, resulting in excessive urinary cystine excretion. Recurrent KSD and increased risk of CKD characterize this condition. Mutations in the transport protein, encoded by *SLC3A1* (Type A) and *SLC7A9* (Type B), accounts for 90% of cases [21]. Our study detected cystinuria gene variants in 5% of patients, including one patient with an undiagnosed autosomal dominant mutation in *SLC7A9*. Due to incomplete penetrance, most heterozygous individuals, including this patient, do not develop cysteine stones [21, 22].

### Study limitations

This study offers valuable insights into the genetic underpinnings of KSD but also has notable limitations. The small sample size resulted in a lower-than-anticipated incidence of pathogenic genetic mutations. The absence of a control group of non-stone formers limits comparative analysis between stone formers and non-stone formers. Focusing exclusively on adults may have excluded relevant genetic mutations found in pediatric KSD cases. The genetic panel we used was not meant for KSD, leading to the detection of unrelated genetic mutations, despite covering many KSD-associated genes. Additionally, only half of the patients completed 24-h urine studies, which may cause biased result interpretation. Nevertheless, Our cohort’s ethnic diversity contrasts with predominantly White populations in other studies.

Genetic testing in KSD presents both opportunities and challenges. It enables personalized medicine by facilitating targeted interventions and preventative measures based on individual genetic profiles. Identifying genetic mutations associated with KSD, such as cystinuria or primary hyperoxaluria, can improve risk assessment and guide early intervention strategies. However, interpreting genetic mutations, particularly VUS, remains challenging and warrants further research. Ethical considerations, including privacy and potential anxiety from uncertain results must also be addressed. Despite these challenges, we believe genetic testing will empower urologists to offer tailored management for KSD patients in the future.

## Conclusions

The clinical utility of genetic testing in KSD offers significant potential for early diagnosis and management, especially in patients with concurrent CKD, complex metabolic profiles, and treatment refractory KSD. Despite challenges in interpreting genetic data, advancements in technology in research promise to enhance diagnostic accuracy and management. Urologists and nephrologists are poised to play a pivotal role in leveraging genetic insights to optimize patient outcomes.

## Supplementary Information

Below is the link to the electronic supplementary material.

Supplementary file1 (DOCX 14 KB)

Supplementary file2 (DOCX 20 KB)

Supplementary file3 (DOCX 110 KB)

Supplementary file4 (DOCX 21 KB)

## Data Availability

The data that support the findings of this study are not openly available due to reasons of sensitivity and are available from the corresponding author upon reasonable request. Data are located in controlled access data storage at Montefiore Medical Center.

## References

[1] Howles SA, Thakker RV (2020) Genetics of kidney stone disease. Nat Rev Urol 17:407–421. 10.1038/s41585-020-0332-x 32533118 10.1038/s41585-020-0332-x

[2] Goldfarb DS, Fischer ME, Keich Y, Goldberg J (2005) A twin study of genetic and dietary influences on nephrolithiasis: A report from the Vietnam Era Twin (VET) Registry. Kidney Int 67:1053–1061. 10.1111/j.1523-1755.2005.00170.x 15698445 10.1111/j.1523-1755.2005.00170.x

[3] Goldfarb DS (2015) The Search for Monogenic Causes of Kidney Stones. J Am Soc Nephrol 26:507–510. 10.1681/ASN.2014090847 25296720 10.1681/ASN.2014090847 PMC4341491

[4] Halbritter J, Baum M, Hynes AM et al (2015) Fourteen Monogenic Genes Account for 15% of Nephrolithiasis/Nephrocalcinosis. J Am Soc Nephrol 26:543–551. 10.1681/ASN.2014040388 25296721 10.1681/ASN.2014040388 PMC4341487

[5] Oddsson A, Sulem P, Helgason H et al (2015) Common and rare variants associated with kidney stones and biochemical traits. Nat Commun 6:7975. 10.1038/ncomms8975 26272126 10.1038/ncomms8975 PMC4557269

[6] Palsson R, Indridason OS, Edvardsson VO, Oddsson A (2019) Genetics of common complex kidney stone disease: insights from genome-wide association studies. Urolithiasis 47:11–21. 10.1007/s00240-018-1094-2 30523390 10.1007/s00240-018-1094-2

[7] Pearle MS, Goldfarb DS, Assimos DG et al (2014) Medical Management of Kidney Stones: AUA Guideline. J Urol 192:316–324. 10.1016/j.juro.2014.05.006 24857648 10.1016/j.juro.2014.05.006

[8] Zeng G, Zhu W, Robertson WG et al (2022) International Alliance of Urolithiasis (IAU) guidelines on the metabolic evaluation and medical management of urolithiasis. Urolithiasis 51:4. 10.1007/s00240-022-01387-2 36454329 10.1007/s00240-022-01387-2

[9] Renasight Overview. In: Natera. https://www.natera.com/organ-health/renasight-genetic-testing/. Accessed 10 Sep 2024

[10] Carpenter TO (2017) CYP24A1 loss of function: Clinical phenotype of monoallelic and biallelic mutations. J Steroid Biochem Mol Biol 173:337–340. 10.1016/j.jsbmb.2017.01.006 28093352 10.1016/j.jsbmb.2017.01.006

[11] Bhadada SK, Sridhar S, Dhiman V et al (2020) Hypophosphatemic rickets with hypercalciuria: a novel homozygous mutation in SLC34a3 and literature review. AACE Clin Case Rep 6:e105–e112. 10.4158/ACCR-2019-0456 32524022 10.4158/ACCR-2019-0456 PMC7282280

[12] Stechman MJ, Loh NY, Thakker RV (2009) Genetic causes of hypercalciuric nephrolithiasis. Pediatr Nephrol 24:2321–2332. 10.1007/s00467-008-0807-0 18446382 10.1007/s00467-008-0807-0 PMC2770137

[13] Gee HY, Jun I, Braun DA et al (2016) Mutations in SLC26A1 Cause Nephrolithiasis. Am J Hum Genet 98:1228–1234. 10.1016/j.ajhg.2016.03.026 27210743 10.1016/j.ajhg.2016.03.026 PMC4908148

[14] Dawson PA, Sim P, Mudge DW, Cowley D (2013) Human SLC26A1 gene variants: a pilot study. ScientificWorldJournal 2013:541710. 10.1155/2013/541710 24250268 10.1155/2013/541710 PMC3819931

[15] Cochat P, Rumsby G (2013) Primary hyperoxaluria. N Engl J Med 369:649–658. 10.1056/NEJMra1301564 23944302 10.1056/NEJMra1301564

[16] Ichida K, Amaya Y, Okamoto K, Nishino T (2012) Mutations Associated with Functional Disorder of Xanthine Oxidoreductase and Hereditary Xanthinuria in Humans. Int J Mol Sci 13:15475–15495. 10.3390/ijms131115475 23203137 10.3390/ijms131115475 PMC3509653

[17] RESERVED IU-AR Orphanet: Hereditary xanthinuria. https://www.orpha.net/consor/cgi-bin/Disease_Search.php?lng=EN&data_id=704&Disease_Disease_Search_diseaseGroup=hereditary-xanthinuria&Disease_Disease_Search_diseaseType=Pat&Disease(s)/group%20of%20diseases=Hereditary-xanthinuria&title=Hereditary%20xanthinuria&. Accessed 12 Feb 2024

[18] Enomoto A, Kimura H, Chairoungdua A et al (2002) Molecular identification of a renal urate–anion exchanger that regulates blood urate levels. Nature 417:447–452. 10.1038/nature742 12024214 10.1038/nature742

[19] Hosoya T, Uchida S, Shibata S et al (2022) Xanthine Oxidoreductase Inhibitors Suppress the Onset of Exercise-Induced AKI in High HPRT Activity Urat1-Uox Double Knockout Mice. J Am Soc Nephrol 33:326–341. 10.1681/ASN.2021050616 34799437 10.1681/ASN.2021050616 PMC8819989

[20] Nakayama A, Matsuo H, Ohtahara A et al (2019) Clinical practice guideline for renal hypouricemia (1st edition). Hum Cell 32:83–87. 10.1007/s13577-019-00239-3 30783949 10.1007/s13577-019-00239-3 PMC6437292

[21] D’Ambrosio V, Capolongo G, Goldfarb D et al (2022) Cystinuria: an update on pathophysiology, genetics, and clinical management. Pediatr Nephrol 37:1705–1711. 10.1007/s00467-021-05342-y 34812923 10.1007/s00467-021-05342-y

[22] Daga S, Palit V, Forster JA et al (2021) An update on evaluation and management in cystinuria. Urology 149:70–75. 10.1016/j.urology.2020.12.025 33421442 10.1016/j.urology.2020.12.025

