# Supplementary material for: Understanding the clinical genetics of kidney stone disease using the Natera Renasight panel
Source: Urolithiasis. 2025 Mar 24;53(1):57. doi: 10.1007/s00240-025-01723-2 (PMC11933196; doi:10.1007/s00240-025-01723-2)
Supplement: Supplementary file 1 — Supplementary file1 (DOCX 14 KB) [file 240_2025_1723_MOESM1_ESM.docx]

## **Appendix 1: *Renasight Kidney Gene Panel (all genes)***

*ABCC6, ABCC8, ABCG2, ACE, ACTB, ADA2, ADAMTS13, ADCY10, AGPAT2, AGT, AGTR1, AGXT, AHI1, ALG1, ALG13, ALG8, ALG9, ALMS1, ALPL, AMN, ANKS6, ANOS1, AP2S1, APOA1, APOC2, APOE, APOL1, APOPT1, APRT, AQP2, ARHGAP24, ARL6, ATP6V0A4, ATP6V1B1, ATP7B, AVP, AVPR2, B2M, BBIP1, BBS1, BBS10, BBS12, BBS2, BBS4, BBS5, BBS7, BBS9, BCS1L, BICC1, BLK, BMP4, BMP7, BMPR2, BRAF, BSCL2, BSND, C5, C8orf37, CA2, CACNA1H, CASR, CAV1, CD151, CD2AP, CDC73, CDKN1C, CEL, CEP164, CEP290, CFH, CFHR5, CFI, CHD1L, CHD7, CHRM3, CHRNA3, CISD2, CLCN2, CLCN5, CLCNKB, CLDN16, CLDN19, CNNM2, COL11A1, COL4A1, COL4A3, COL4A4, COL4A5, COQ2, COQ6, COX10, COX14, COX20, COX6B1, COX8A, CPLANE1, CPT2, CREBBP, CRKL, CTNS, CUBN, CUL3, CYP11A1, CYP11B1, CYP11B2, CYP17A1, CYP24A1, CYP27B1, CYP2R1, DCDC2, DGKE, DHCR7, DLC1, DMP1, DNASE1L3, EBP, EDNRA, EGF, EIF2AK3, ELP1, ENPP1, EYA1, FAM20A, FAN1, FANCA, FANCB, FANCC, FANCD2, FANCE, FANCF, FANCG, FANCI, FANCL, FANCM, FASTKD2, FGA, FGF10, FGF23, FGFR1, FGFR2, FLCN, FN1, FOXC1, FOXC2, FOXI1, FOXP3, FRAS1, FREM1, FREM2, FXYD2, G6PC, GALNT3, GANAB, GATA3, GATM, GCK, GCM2, GDNF, GLA, GLI3, GLIS2, GLIS3, GNA11, GNAS, GPC3, GREM1, GRHPR, GRIP1, GSN, HBB, HGD, HNF1A, HNF1B, HNF4A, HOGA1, HOXA13, HOXD13, HPRT1, HPS1, HPSE2, HSD11B2, HSD3B2, IFT122, IFT140, IFT172, IFT43, INF2, INS, INVS, IQCB1, ITGA3, ITGA6, ITGB4, ITSN2, JAG1, KANK1, KANSL1, KAT6B, KCNA1, KCNJ1, KCNJ10, KCNJ11, KCNJ5, KCNK3, KCTD1, KL, KLF11, KLHL3, KRAS, LAMB2, LCAT, LDHA, LMNA, LMX1B, LPIN1, LRP2, LRP4, LRP5, LYZ, LZTFL1, MAFB, MAGI2, MC4R, MEFV, MKKS, MMACHC, MNX1, MOCOS, MUT, MVK, MYCN, MYH9, MYO1E, NEDD4L, NEK8, NEUROD1, NF1, NLRP3, NOTCH2, NPHP1, NPHP3, NPHP4, NPHS1, NPHS2, NR0B1, NR3C1, NR3C2, NSD1, NSDHL, OCRL, OFD1, OPLAH, PALB2, PAX2, PAX4, PBX1, PCBD1, PDSS1, PDSS2, PDX1, PET100, PGK1, PHEX, PKD1, PKD2, PKHD1, PLCE1, PLCG2, PLG, PMM2, PPP3CA, PRKCSH, PRODH, PROKR2, PRPS1, PTH1R, PTPN11, PTPRO, RAD51C, REN, RET, RMND1, ROBO2, ROR2, RPGRIP1L, RPL11, RPL26, RPL35A, RPL5, RPS10, RPS17, RPS19, RPS24, RPS26, RPS7, RRM2B, SALL1, SALL4, SARS2, SCARB2, SCN4A, SCNN1A, SCNN1B, SCNN1G, SCO1, SDCCAG8, SEC63, SEMA3E, SI, SIX1, SIX2, SIX5, SLC12A1, SLC12A2, SLC12A3, SLC16A12, SLC22A12, SLC26A1, SLC2A2, SLC2A9, SLC34A1, SLC34A3, SLC36A2, SLC37A4, SLC3A1, SLC41A1, SLC4A1, SLC4A4, SLC5A1, SLC5A2, SLC6A19, SLC6A20, SLC7A7, SLC7A9, SLC9A3R1, SLX4, SMAD9, SMARCAL1, SMC1A, SOX17, SOX18, SRCAP, STAR, STK39, STX16, SYNPO, TACO1, TFAP2A, THBD, TMEM67, TNS2, TP53RK, TP63, TRIM32, TRPC6, TRPM6, TSC1, TSC2, TTC21B, TTC8, TTR, TXNL4A, UCP3, UMOD, UPK3A, UQCC2, VDR, VHL, WAS, WDPCP, WDR19, WDR72, WDR73, WFS1, WNK1, WNK4, WNT4, WNT5A, WT1, XDH, XPNPEP3, XRCC4.*
