# Supplementary material for: Understanding the clinical genetics of kidney stone disease using the Natera Renasight panel
Source: Urolithiasis. 2025 Mar 24;53(1):57. doi: 10.1007/s00240-025-01723-2 (PMC11933196; doi:10.1007/s00240-025-01723-2)
Supplement: Supplementary file 2 — Supplementary file2 (DOCX 20 KB) [file 240_2025_1723_MOESM2_ESM.docx]

## **Appendix 2: *Renasight Kidney Gene Panel (******Kidney Stone Disease related genes and conditions)***

| **Gene** | **Condition** | **Autosomal Recessive (AR), Autosomal Dominant (AD), or other** |
| --- | --- | --- |
| *AGXT* | Primary Hyperoxaluria Type 1 (AR) | AR |
| *GRHPR* | Hyperoxaluria, Primary, Type 2 (AR) | AR |
| *HOGA1* | Primary Hyperoxaluria, Type 3 (AR) | AR |
| *SLC3A1* | Cystinuria (AD/AR) | AD (certain variants)/AR |
| *SLC7A9* | Cystinuria (AD/AR) | AD (certain variants)/AR |
| *CLDN16* | Hypomagnesemia 3, Renal (AR) | AR |
| *CLDN19* | Hypomagnesemia 5, Renal, with Ocular Involvement (AR) | AR |
| *XDH* | Xanthinuria, Type I (AR) | AR |
| *MOCOS* | Xanthinuria, Type II | AR |
| *MOCS1* | Molybdenum Cofactor Deficiency A (AR) | AR |
| *MOCS2* | Molybdenum Cofactor Deficiency B (AR) | AR |
| *ALPL* | hypophosphatasia | AD (certain variants)/AR |
| *APRT* | adenine phosphoribosyltransferase deficiency | AR |
| *ATP7B* | Wilson disease | AR |
| *CASR* | CASR-related conditions (AD/AR), Neonatal hyperparathyroidism (NSHPT) (AR), Hypocalcemia type 1 (ADH1) with or without Bartter syndrome and Familial Hypocalciuric Hypercalcemia type 1 (FHH1) (AD) | AD (certain variants)/AR |
| *CYP24A1* | CYP24A1-related hypercalcemia (AR) | AD limited evidence / AR |
| *FAM20A* | Amelogenesis Imperfecta, Type 1G (AR) | AR |
| *HPRT1* | HPRT1-Related Disorders (XL), Lesch-Nyhan Syndrome (LNS), HPRT1-related neurologic dysfunction, and HPRT1-related hyperuricemia (HRH) | Females can be symptomatic but can develop gout later in life |
| *MOCOS* | Xanthinuria, Type II | AR |
| *SLC22A12* | Hypouricemia, Renal 1 (AR) | AR |
| *SLC26A1* | Nephrolithiasis, Calcium Oxalate (AR) | AR |
| *SLC34A1* | Hypophosphatemic Nephrolithiasis/Osteoporosis 1 (AD) & Infantile Hypercalcemia 2 (AR) | AD/AR |
| *OPLAH* | 5-Oxoprolinase Deficiency (AR) | AR |
| *GNA11* | Autosomal Dominant Hypocalcemia 2 (AD); Hypocalciuric hypercalcemia, type II (AD) | AD (certain variants) |
| *SLC2A9* | Renal Hypouricemia 2 (AD/AR) | AD (certain variants)/AR |
| *PTH1R* | Metaphyseal Chondrodysplasia, Murk Jansen Type (AD); Failure Of Tooth Eruption, Primary (AD); Chondrodysplasia, Blomstrand Type (AR); Eiken Syndrome (AR) | Metaphyseal Chondrodysplasia, Murk Jansen Type (AD) |
| *ABCC6* | Pseudoxanthoma Elasticum (PXE) and Generalized Arterial Calcification of Infancy (GACI) | AD limited evidence / AR |
| *SI* | Sucrase-Isomaltase Deficiency (AR) | AR |
| *HGD* | Alkaptonuria (AR) | AR |
| *AP2S1* | Familial Hypocalciuric Hypercalcemia, Type 3 (AD) | AD |
| *ATP6V0A4* | ATP6V0A4-Distal Renal Tubular Acidosis (AR) | AR |
| *ATP6V1B1* | ATP6V1B1-distal renal tubular acidosis (ATP6V1B1-dRTA) (AR) | AD limited evidence / AR |
| *SLC12A1* | Bartter syndrome type I | AR |
| *CLCNKB* | Bartter Syndrome, Type 3/4B (AR); Gitelman syndrome (AR) | AR |
| *KCNJ1* | Bartter syndrome type 2 (AR) | AR |
| *CDC73* | familial isolated hyperparathyroidism, hyperparathyroidism-jaw tumor syndrome (HPT-JT), and parathyroid carcinoma | AD |
| *GCM2* | Familial Isolated Hypoparathyroidism (AD/AR); Familial Isolated Hyperparathyroidism (AD) | AD (certain variants) |
| *CLCN5* | Dent disease 1 (XL) | Females can be symptomatic |
| *OCRL* | Dent disease 2 and Lowe syndrome (XL) | Females can be symptomatic |
| *VDR* | Vitamin D-Dependent Rickets, type 2A | AR |
| *SLC34A3* | hypophosphatemic rickets with hypercalciuria (AR) | AD limited evidence / AR |
| *CA2* | osteopetrosis with renal tubular acidosis (RTA) (AR) |  |
| *AGPAT2* | lipodystrophy congenital generalized, type 1 (AR) | AR |
| *KLHL3* | Pseudohypoaldosteronism, Type 2D (AD/AR) | AD/AR |

AD = Autosomal Dominant, AR = Autosomal Recessive, XL = X-Linked
