# Supplementary material for: Understanding the clinical genetics of kidney stone disease using the Natera Renasight panel
Source: Urolithiasis. 2025 Mar 24;53(1):57. doi: 10.1007/s00240-025-01723-2 (PMC11933196; doi:10.1007/s00240-025-01723-2)

## **SUPPLEMENTARY FIGURE 1: Genetic Frequency among 105 Kidney Stone Disease patients on Natera Renasight Panel (all genes)**


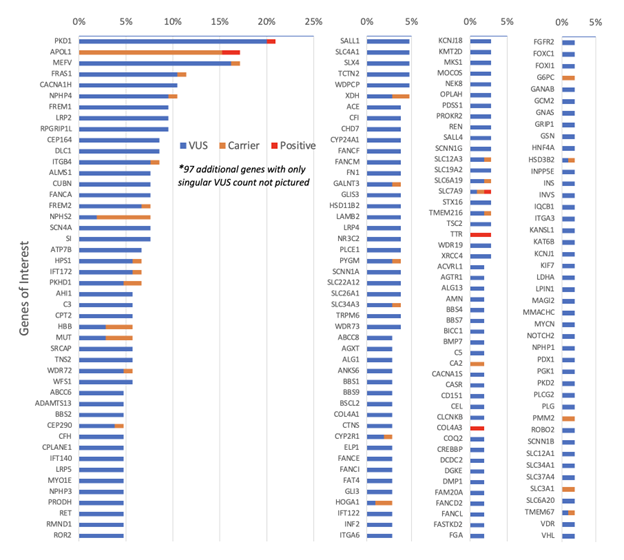

Supplement: Supplementary file 3 — Supplementary file3 (DOCX 110 KB) [file 240_2025_1723_MOESM3_ESM.docx]
