# Supplementary material for: Understanding the clinical genetics of kidney stone disease using the Natera Renasight panel
Source: Urolithiasis. 2025 Mar 24;53(1):57. doi: 10.1007/s00240-025-01723-2 (PMC11933196; doi:10.1007/s00240-025-01723-2)
Supplement: Supplementary file 4 — Supplementary file4 (DOCX 21 KB) [file 240_2025_1723_MOESM4_ESM.docx]

**Supplementary Table 1: 24-hour urine study parameters, stratified by KSD gene groups**

|  | **Total (N=111)** | **Calcium Genes (N=29)** | **p-Value** | **Uric Acid Genes (N=11)** | **p-Value** | **Cysteine Genes (N=5)** | **p-Value** | **Oxalate Genes (N=7)** | **p-Value** |
| --- | --- | --- | --- | --- | --- | --- | --- | --- | --- |
| 24-hour urine study completed | 54 (49%) | 16 (55%) | 0.41 | 4 (36%) | 0.39 | 2 (40%) | 0.69 | 2 (29%) | 0.27 |
| Volume (Liters),  Mean (SD) | 1.80 (±0.90) | 1.92 (±1.13) | 0.56 | 1.43 (±0.29) | 0.39 | 3.02 (±1.12) | **0.05** | 1.48 (±0.36) | 0.60 |
| Calcium,  Mean (SD) | 163 (±85) | 155 (±91) | 0.66 | 99 (±30) | 0.12 | 178 (±52) | 0.81 | 116 (±11) | 0.42 |
| Creatinine,  Mean (SD) | 1488 (±446) | 1598 (±533) | 0.24 | 1315 (±213) | 0.42 | 1438 (±137) | 0.87 | 1517 (±171) | 0.93 |
| Phosphorus, Mean (SD) | 0.782 (±0.273) | 0.837 (±0.283) | 0.35 | 0.715 (±0.277) | 0.61 | 0.637 (±0.116) | 0.45 | 0.780 (±0.378) | 0.99 |
| Urine pH,  Mean (SD) | 6.10 (±0.54) | 6.10 (±0.53) | 1.00 | 5.95 (±0.46) | 0.57 | 6.25 (±0.55) | 0.70 | 6.14 (±0.59) | 0.92 |
| Sodium,  Mean (SD) | 153 (±71) | 152 (±69) | 0.98 | 135 (±26) | 0.61 | 97 (±35) | 0.26 | 166 (±17) | 0.79 |
| Urea Nitrogen,  Mean (SD) | 9.32 (±3.65) | 9.87 (±3.98) | 0.47 | 8.53 (±2.85) | 0.66 | 6.30 (±0.94) | 0.24 | 9.71 (±1.78) | 0.88 |
| Oxalate,  Mean (SD) | 33 (±13) | 32 (±11) | 0.55 | 26 (±4) | 0.22 | 30 (±11) | 0.67 | 28 (±8) | 0.52 |
| Chloride,  Mean (SD) | 152 (±73) | 146 (±69) | 0.74 | 123 (±25) | 0.42 | 103 (±35) | 0.34 | 178 (±34) | 0.61 |
| Ammonium,  Mean (SD) | 32 (±12) | 36 (±12) | 0.11 | 24 (±3) | 0.18 | 32 (±8) | 0.96 | 31 (±7) | 0.91 |
| Magnesium,  Mean (SD) | 83 (±41) | 88 (±35) | 0.57 | 81 (±29) | 0.93 | 73 (±8) | 0.73 | 103 (±6) | 0.48 |
| Potassium,  Mean (SD) | 54 (±27) | 53 (±23) | 0.84 | 52 (±11) | 0.86 | 54 (±13) | 0.99 | 70 (±23) | 0.40 |
| Uric Acid,  Mean (SD) | 0.668 (±0.349) | 0.752 (±0.521) | 0.26 | 0.464 (±0.112) | 0.23 | 0.603 (±0.253) | 0.79 | 0.664 (±0.139) | 0.99 |
| Sulfate,  Mean (SD) | 34 (±15) | 38 (±16) | 0.15 | 35 (±13) | 0.87 | 21 (±0) | 0.23 | 36 (±3) | 0.82 |
| Citrate,  Mean (SD) | 506 (±317) | 617 (±353) | 0.09 | 757 (±435) | 0.10 | 844 (±194) | 0.13 | 330 (±42) | 0.43 |

SD = Standard Deviation
